# Supplementary material for: Connectivity and function are coupled across cognitive domains throughout the brain
Source: Netw Neurosci. 2026 Jan 8;10(1):80–92. doi: 10.1162/NETN.a.504 (PMC12798649; doi:10.1162/NETN.a.504)

Supplemental materials for: Connectivity and function are coupled across cognitive domains throughout the brain**;** Kelly J. Hiersche, Zeynep M. Saygin, David E. Osher

Figure S1: Heatmap of model fit for all domains across lobes. Regions are in the same order as presented in Figure 2.

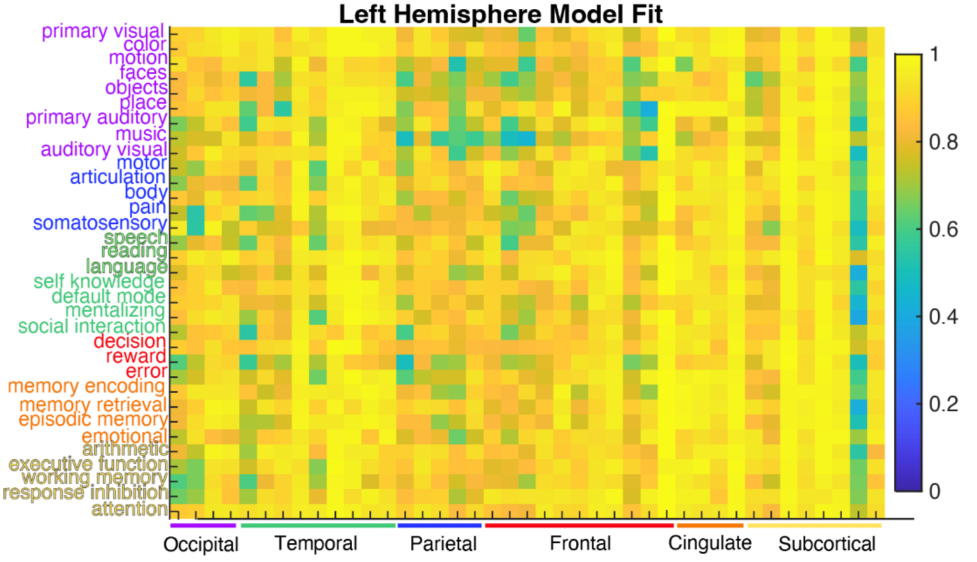

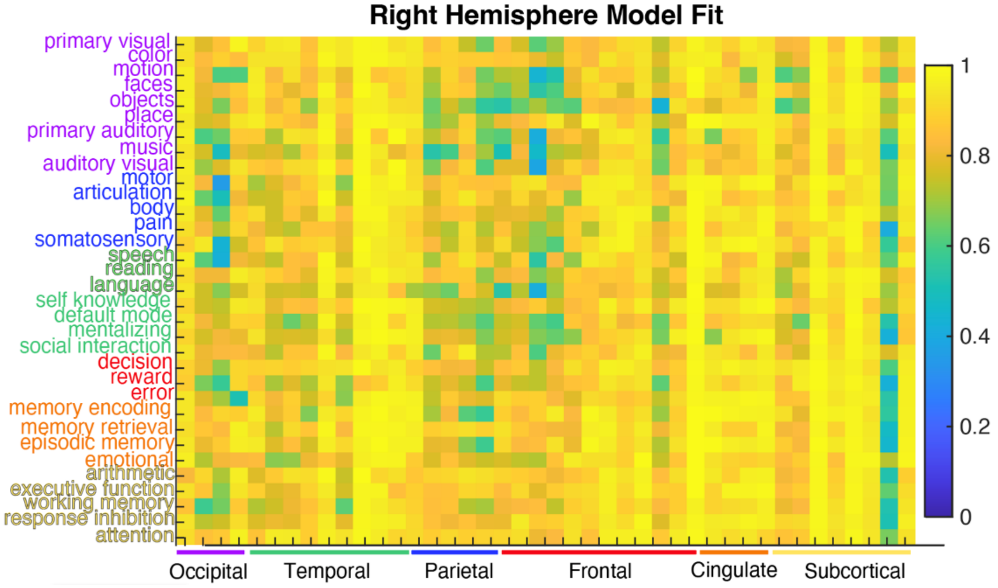

Figure S2: NeuroQuery expected activation maps (used as outcome variable in models) for all 33 cognitive domains on left lateral surface of FsAverage.

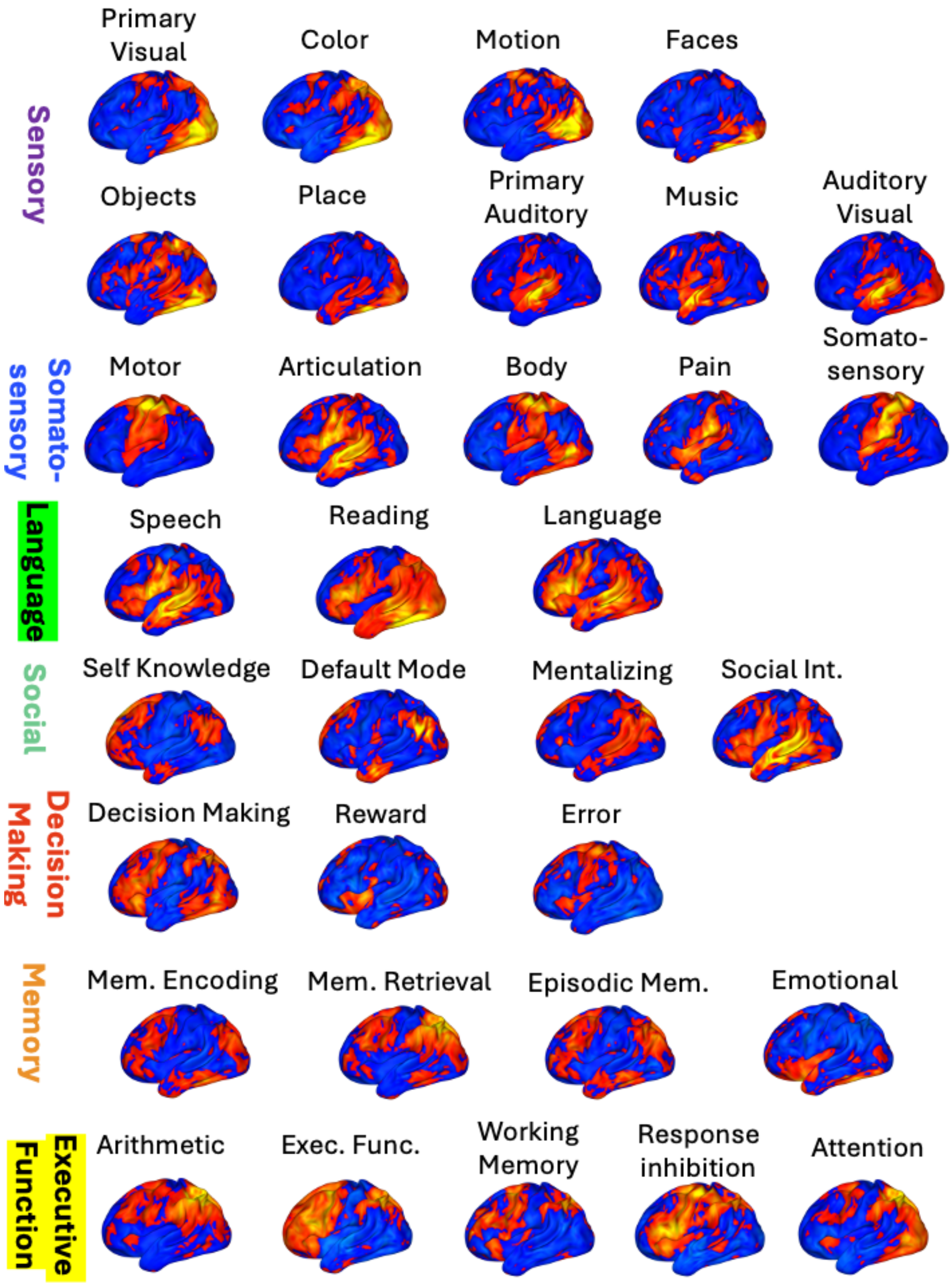

Supplement: Supplementary file 1 [file netn-10-1-80-s001.pdf]
